# Supplementary material for: A Change in Conservation Status of Pachyphytum caesium (Crassulaceae), a Threatened Species from Central Mexico Based on Genetic Studies
Source: Biology (Basel). 2022 Feb 27;11(3):379. doi: 10.3390/biology11030379 (PMC8945335; doi:10.3390/biology11030379)
Supplement: Supplementary file 1 [file biology-11-00379-s001.zip › biology-1569029-supplementary.pdf]

Table S1. Details of screened ISSR primers tested in the study of *Pachyphytum caesium*. In bold ISSR discarded

Primer ID	Primer sequence (5´-3´)	Number of loci detected	Annealing temperature (°C)
814	CTC TCT CTC TCT CTCTTC	6	48
844A	CTC TCT CTC TCT CTC TAG	6	48
**844B**	CTC TCT CTC TCT CTC TGC	0*	54+
17898A	CAC AGA CAC AGA CT	10	37
17899A	GAC AGA GAG AGA CT	9	40
17899B	CAC ACA CAC AGA AC	8	37
17899B-1	GAC AGA GAG AGA AC	8	40
HB8	GAC AGA GAG AGA GG	7	40
**HB9**	GTC TGT GCT TGC GG	0*	40
**HB10**	GAG AGA GAG AGA GG	0*	40
HB11	GTC TGC GTC TGC GG	10	40
HB12	CAC CAC AGC C	10	37
HB13	GAC GAG GAG GC	9	38
HB14	CTC CTC CTC GC	9	38
HB15	GTC GTC GTC GC	9	38

*Primers were not select, annealing temperature (°C) was standardizing for each primer before the analysis; + The PCR reaction of this primer was carried at annealing with 4 °C out than the melting temperature calculated by the manufactured.

Table S2. PCR reaction used in the study of *Pachyphytum caesium*.

Reagent	Volume
Distilled water (PiSA, Mexico)	15.63 μl
Master Stock*	5.07 μl (2.16 μl of purified water, 2.6 μl of 1X buffer (pH 8.4); 0.05 μl of $MgCl_2$ (1 M); and 0.26 μl (0.1 mM) of each dNTPs
Primer-50μM (Invitrogen, Scientific, United States)	2 μl
Taq DNA 1.5 U (Invitrogen, Scientific, United States)	0.3μl
DNA template 10ng	2μl
**Total**	25μl

*See Table S3 for the preparation of the Master stock.

Table S3. Preparation of MASTER STOCK used in the study of *Pachyphytum caesium*.

Reagent	Volume
Distilled water (PiSA, Mexico).	265 μl
Buffer of PCR 1X pH=8.4 (Invitrogen, Fisher Scientific, United States).	500 μl
$MgCl_2$ 1M (Sigma)	10 μl
DNTP´s (Fisher Scientific, United States)	200 μl (1:10) *
TOTAL	975 μl

*1:10 in DNTP´s (dATP, dCTP, dTTP and dGTP), is equal to 10μl of each one of DNTP´s in 60μl of water

Table S4. PCR program used in the study of *Pachyphytum caesium*.

Cycle steps	Temperature (°C)	Time
Initial denaturation	94	5 min
1	94	1 min (denaturation)
2	38-54 (T_m)	1:30 min (annealing)
3	72	2 min (extension)
Number of cycles	44	4:20 h:min
Final extension	72	13 min
Hold	4	hold temperature of 4°C at the end

T_m = specific annealing temperature of each primer

Table S5. Genetic diversity of the six studied populations of *Pachyphytum caesium*. N = number of individuals per population; Nx = number of individuals per population simulating loss of individuals; N_{50} = number of individuals simulating local populations extinction; Io = number of total alleles; Iox = number of total allele with local extinction; I_{50} = number of total allele with loss of individuals; Ie = number of private alleles; Iex = number of private alleles with local extinction; Ie_{50} = number of private alleles with loss of individuals; Eff_num = effective number of alleles; Eff_numx = effective number of alleles with local extinction; Eff_num_{50} = effective number of alleles with loss of individuals; He = expected heterozygosity; Hex = expected heterozygosity with local extinction; He_{50} = expected heterozygosity with loss of individuals; %P = percentage of polymorphic; %Px = percentage of polymorphic with local extinction; $\%P_{50}$ = percentage of polymorphic with loss of individuals; I = Shannon index; Ix = Shannon index with local extinction; Is_{50} = Shannon index with loss of individuals.

	**Presa Malpaso**	**Puente Cuates**	**Río Gil**	**Barrancas Tortugas**	**Presa Cebolletas**	**Mesa Montoro**	**Average**
N	20	20	20	20	20	20	20
Nx	20	20	20	20	0	0	20
N_{50}	10	10	10	10	10	10	10
I_o	85	90	91	90	96	86	89.7±3.9
I_{ox}	85	90	91	90	0	0	89.0±2.7
I_{50}	82	82	90	90	94	86	87.7±5.9
I_e	0	1	0	0	4	0	0.83±1.7
I_{ex}	0	1	0	0	0	0	0.25±0.5
I_{e50}	0	0	0	0	1	0	0.17±0.3
Eff_num	1.3	1.242	1.263	1.222	1.333	1.733	1.34±0.19
Eff_num_x	1.268	1.185	1.212	1.229	0	0	1.22±0.06
Eff_num_{50}	1.181	1.123	1.2	0	0	1.122	0.77±0.6
H_e	0.186	0.188	0.221	0.197	0.297	0.187	0.212±0.0
H_{ex}	0.186	0.188	0.221	0.197	0	0	0.198±0.0
H_{e50}	0.147	0.141	0.191	0.187	0.262	0.142	0.178±0.05
%P	48.51	55.45	55.45	55.43	73.27	46.53	56.1±3.9
%Px	48.51	55.45	57.43	55.43	0.00	0.00	54.21 ±1.3
$\%P_{50}$	36.63	38.61	46.53	55.45	66.34	34.37	46.3±5.1
I	0.272	0.283	0.321	0.296	0.398	0.272	0.307±0.0
I_{ix}	0.272	0.283	0.321	0.296	0	0	0.295±0.0
I_{i50}	0.213	0.208	0.276	0.293	0.381	0.142	0.252±0.01

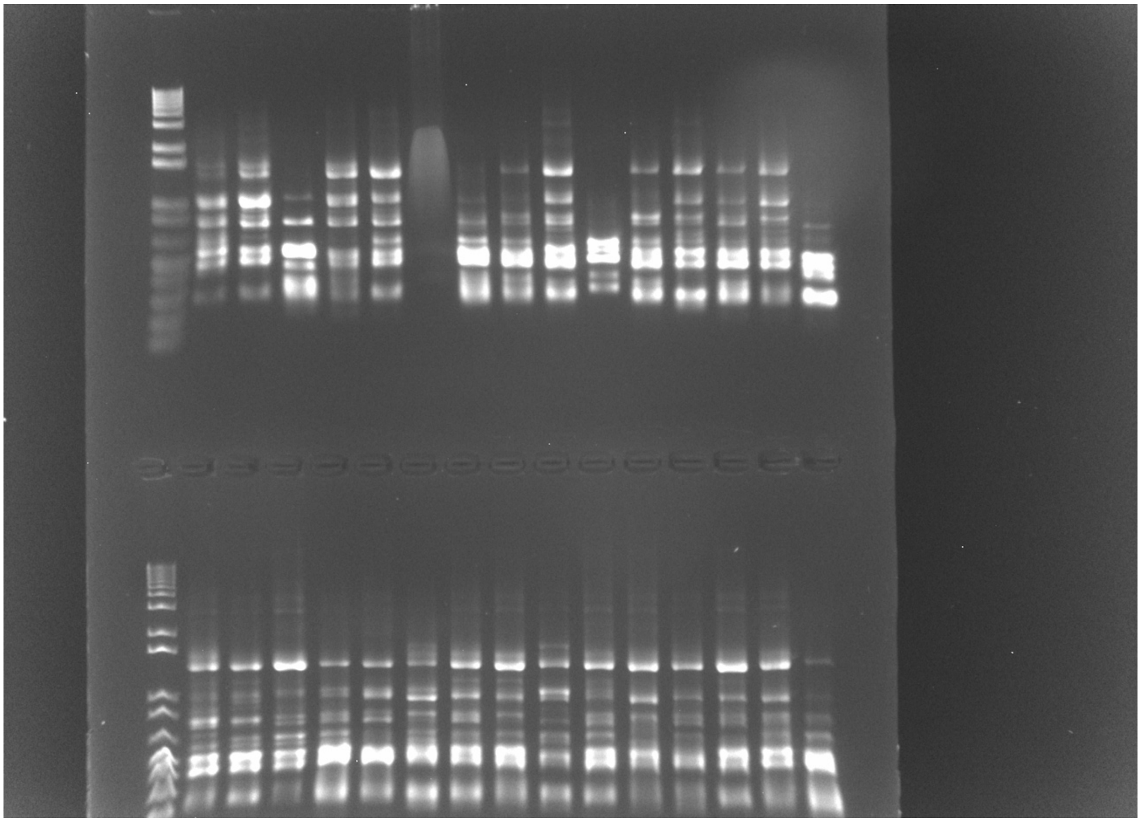

Figure S1. Amplification profile of *P. caesium* samples of Puente Cuates and Rio Gil population using 17898A primer.
